# Development of a novel cell-based assay system EPISSAY for screening epigenetic drugs and liposome formulated decitabine

**DOI:** 10.1186/1471-2407-13-113

**Published:** 2013-03-13

**Authors:** Sue Ping Lim, Raman Kumar, Yamini Akkamsetty, Wen Wang, Kristen Ho, Paul M Neilsen, Diego J Walther, Rachel J Suetani, Clive Prestidge, David F Callen

**Affiliations:** 1 Cancer Therapeutics Laboratory, Centre for Personalized Cancer Medicine, The University of Adelaide, Adelaide, South Australia, Australia; 2 Women’s & Children’s Health Research Institute Inc, North Adelaide, South Australia, Australia; 3 Ian Wark Research Institute, University of South Australia, Mawson Lakes Boulevard, Mawson Lakes, South Australia, Australia; 4 Max Planck Institute for Molecular Genetics, Berlin, Germany

**Keywords:** Cell-based assay system, Decitabine, Liposomes, Nanotechnology, CB1954, Nitroreductase

## Abstract

**Background:**

Despite the potential of improving the delivery of epigenetic drugs, the subsequent assessment of changes in their epigenetic activity is largely dependent on the availability of a suitable and rapid screening bioassay. Here, we describe a cell-based assay system for screening gene reactivation.

**Methods:**

A cell-based assay system (EPISSAY) was designed based on a silenced triple-mutated bacterial nitroreductase TMnfsB fused with Red-Fluorescent Protein (RFP) expressed in the non-malignant human breast cell line MCF10A. EPISSAY was validated using the target gene *TXNIP*, which has previously been shown to respond to epigenetic drugs. The potency of a epigenetic drug model, decitabine, formulated with PEGylated liposomes was also validated using this assay system.

**Results:**

Following treatment with DNA methyltransferase (DNMT) and histone deacetylase (HDAC) inhibitors such as decitabine and vorinostat, increases in RFP expression were observed, indicating expression of *RFP-TMnfsB*. The EPISSAY system was then used to test the potency of decitabine, before and after PEGylated liposomal encapsulation. We observed a 50% higher potency of decitabine when encapsulated in PEGylated liposomes, which is likely to be due to its protection from rapid degradation.

**Conclusions:**

The EPISSAY bioassay system provides a novel and rapid system to compare the efficiencies of existing and newly formulated drugs that reactivate gene expression.

## Background

DNA methylation and histone modification are the two major epigenetic mechanisms catalyzed by DNMTs and HDACs, respectively [1]. HDACs remove the acetyl groups from histones, whilst DNMTs catalyse the transfer of a methyl group from S-adenosylmethionine to the 5-carbon position of the cytosine pyrimidine ring, both leading to the condensation of chromatin to its inactive state [2,3]. In cancer cells, an abundance of hypo-acetylated histones is usually associated with DNA hyper-methylation and gene silencing [4]. These findings are the basis for the development of HDAC and DNMT inhibitors as cancer therapeutics. Such compounds block the activity of HDACs and DNMTs, leading to increased expression of epigenetically silenced genes which mediate cellular and metabolic changes such as cell growth arrest, differentiation and apoptosis [5-9].

Hydrophobic vorinostat (suberoylanilide hydroxamic acid, SAHA) and hydrophilic decitabine (5-aza-2^′^-deoxycytidine, Dacogen) are US Food and Drug Administration (FDA) approved HDAC and DNMT inhibitors for the treatment of cutaneous T-cell lymphoma and myelodysplastic syndrome, respectively [10,11]. The combination of vorinostat and decitabine have been shown to have promising activity in patients with myelodysplastic syndrome without significant toxicity in a phase I clinical trial [12]. Under neutral conditions, decitabine has a reported half-life of 7 days at 4°C or 21 hours at 37°C *in vitro*[13]. However, decitabine is degraded more rapidly *in vivo* with a half-life of only 25 minutes [13]. Such chemical instability of decitabine has led to its administration in the clinic as a cold and continuous intravenous infusion in an effort to reach the maximal-tolerated doses required to achieve clinical response [14,15].

The development of drug formulation using nanotechnology (e.g. liposomes) has been used to improve drug stability [16,17]. Despite the potential of improving the delivery of epigenetic drugs, the subsequent assessment of changes in their epigenetic activity is largely dependent on the availability of a suitable and rapid screening bioassay. A commonly used cell-based assay for both DNMT and HDAC inhibitors is the quantification of the re-expression of known epigenetically-silenced genes by reverse transcription polymerase chain reaction (RT-PCR) and western blot analysis [5,18]. However, this traditional approach is not high-throughput and may produce gene-specific results. Other assays that have been used include estimation of global DNA methylation using capillary electrophoresis, DNA digestion with methylation-sensitive restriction enzymes, or analysis of specific DNA methylation using bisulfite sequencing and methylation-specific PCR [19]. However, these assay systems designated for assaying DNMT or HDAC inhibitors are time-consuming, cumbersome and subject to misinterpretation [20-22]. Consequently, the rapid identification and validation of novel epigenetic drugs are hampered due to the lack of an efficient screening method.

In this study, a cell-based assay system was developed to compare the activity of different epigenetic drugs. This assay system is based on mammalian MCF10A cells expressing a fusion protein between red-fluorescent protein (RFP) and bacterial nitroreductase (TMnfsB) driven by CMV promoter. Epigenetic silencing has been shown to silence genes driven by CMV promoter in both stably transfected cells and transgenic pigs [23,24]. Silenced CMV promoter driven genes were shown to be reactivated after treatment with epigenetic drugs such as butyrate, trichostatin A and decitabine [23]. Human cells expressing TMnfsB are able to metabolize the monofunctional alkylating prodrug CB1954 (5-(azaridin-1-yl)-2,4-dinitro-benzamide) to highly cytotoxic hydroxylamino- and amino-derivatives, which induce rapid cell death [25]. Therefore, TMnfsB was utilized as a tool to obtain clones with inactivated CMV promoters. The *TMnfsB* open reading frame has been codon optimized to increase the sensitivity of stable human cell lines to the prodrug CB1954 [26]. An assay system for gene reactivation was developed by identifying clones where expression of RFP-TMnfsB was suppressed at the transcriptional level, but could be re-established by subsequent treatment with epigenetic drugs. Since RFP expression in these clones is low, it was used as a signal to evaluate the reactivation of gene expression by flow cytometry. Using this newly developed assay system, it was shown that decitabine which encapsulated in the liposomes has a higher gene restoring ability than pure decitabine, zebularine and RG108.

## Methods

### Plasmids

The mammalianized nitroreductase gene B (*TMnfsB*) vector was generated by subcloning the nitroreductase open reading frame from existing constructs kindly provided by Grohmann et al. [26] into the pDsRED-C1-monomer vector at a *Xho*I/*Bam*HI site. A retroviral plasmid pLNCX2-*RFP-TMnfsB* expressing RFP-TMnfsB fusion was generated by subcloning the *RFP-TMnfsB* coding fragment from the existing construct pDsRED-*TMnfsB* (*Sna*BI/*Bam*HI) into the pLNCX2 vector (*Sna*BI/*Bgl*II). All constructs were confirmed by sequencing using appropriate primers (Additional file 1).

### Cell culture

All human cell lines were purchased from the American Type Culture Collection (ATCC) except the Phoenix retrovirus producer cell line which was kindly provided by Prof. Garry Nolan of Stanford University (United States). All cell lines were grown in the ATCC recommended media.

### Reagents

CB1954 (soluble to 2 mg/mL in aqueous solution), decitabine (soluble to 50 mg/mL in aqueous solution), 2(1H)-pyrimidinone riboside (zebularine; soluble to 16 mg/mL in DMSO) and RG108 (soluble to 10 mg/mL in DMSO) were purchased from Sigma. RG108 is known to be an ineffective DNMT inhibitor [27] and was used as a negative control. Vorinostat (10 mM) was kindly supplied by Dr. Lisa Butler of The University of Adelaide (South Australia). All drugs were dissolved in DMSO except decitabine, which was prepared in water for liposomal formulation. The synthetic lipids 1,2-dioleoyl-sn-glycero-3-[phospho-rac-(1-glycerol)] sodium salt (DOPG), 1,2 distearoyl-sn-glycero-3-phosphocholine (DSPC), 1,2-distearoyl-sn-glycero-3-phosphoethanolamine-N-[amino(polyethylene glycol)-2000] ammonium salt (DSPE-PEG2000) and natural cholesterol lipid were purchased from Avanti Polar Lipids.

### Generation of stable cell line and clonal selection

Recombinant retrovirus encoding RFP-TMnfsB was produced using the Phoenix packaging cell line transfected with Lipofectamine 2000 (Invitrogen) according to the recommended protocol. Stable cell lines expressing RFP-TMnfsB were generated by G418 selection of MCF10A cells transduced with retrovirus expressing RFP-TMnfsB for approximately 2 months. G418-resistant MCF10A cells were grown into colonies in 10 cm dishes and potential clones where TMnfsB was spontaneously silenced were isolated by treating these colonies with 5 μM of CB1954 for 72 hours. Surviving colonies, which were potentially epigenetically silenced, were isolated as CB1954-resistant clones. The integrity of *RFP-TMnfsB* in CB1954-resistant clones was determined by screening using RT-PCR. Finally, colonies with silenced *RFP-TMnfsB* insert were identified by assessing TMnfsB and RFP expression using RT-PCR and flow cytometry, respectively, after treatment with epigenetic drugs.

### Real-time polymerase chain reaction (RT-PCR)

RNA and DNA from the cells were extracted using the RNeasy plant mini kit (Qiagen) and the DNeasy Blood and Tissue Kit (Qiagen), respectively. cDNA was generated using random primers and 20 U of reverse transcriptase (Promega). *TXNIP*, *TMnfsB* and *RFP-TMnfsB* expression were determined by qRT-PCR using IQ™ SYBR green supermix (Biorad) and primers listed in Additional file 1. Cycling conditions were: 10 min at 95°C followed by 40 repeats of 95°C for 10 s, annealing at appropriate temperature for 15 s and extension at 72°C for 10 s. β-actin expression was used for normalization of target gene expression.

### Western blotting

Western blot analysis of RFP-TMnfsB fusion protein expressed in MCF10A cells was performed using a rabbit polyclonal anti-RFP antibody (Invitrogen) or mouse anti-β-actin antibody (Sigma-Aldrich), and a secondary donkey anti-rabbit IgG-HRP (GE Healthcare) or a sheep anti-mouse IgG-HRP (GE Healthcare) [28]. Total cellular proteins were extracted as described previously [29] and visualized by an Enhanced Chemiluminescence Detection Kit (Amersham Biosciences).

### Flow cytometry

The reactivation of silenced RFP-TMnfsB was determined by flow cytometry. Cells were plated at 40% 24 hours prior to treatment. The approximate doubling time of the cells is 48 hours. Cells were treated with each drug (decitabine 1, 5, 10, 30 and 50 μM; zebularine 50, 100, 250 and 500 μM; RG108 10 and 100 μM; vorinostat 1 and 2 μM) for 48 or 72 hours in triplicate. The red-fluorescence of cells was analyzed at a log scale of geometric mean of FL3-H using FACSCalibur flow cytometer (BD). Data were processed using WinMDI v2.8 software.

### Preparation of liposomal decitabine

Liposomal formulations were prepared according to the method developed by Sunoqrot and colleagues with minor modifications [30]. Briefly, 5 mg (32.5 mol%) DOPG, 4.9 mg (32.1 mol%) DSPC, 1.8 mg (3.3 mol%) DSPE-PEG2000 and 2.4 mg (32.1 mol%) cholesterol were dissolved in 5 mL of chloroform. Thin lipid films were generated after removing the solvent in a rotary evaporator for 2 hours at room temperature. Liposomes were formed when thin lipid films (4 mM) were hydrated in 5 mL of water or 0.88 mM decitabine dissolved in water for 1 hour at room temperature and stored at 4°C. The samples were extruded ten times using 200 and 400 nm polycarbonate membranes to obtain unilamellar liposomes.

### High performance liquid chromatography (HPLC)

HPLC (Shimadzu LC-10AT) analysis was done using a XTerraTM C8 analytical column at 254 nm, using MiliQ water as mobile phase and a flow rate of 0.8 mL/min. The limit of quantification of decitabine is 10 ng/mL [31,32].

### Liposomes characterization

The size and zeta potential of liposomes were characterized by dynamic laser light scattering (Malvern Zetasizer Nanoseries). Data are expressed as the mean plus standard deviation of three technical repetitive measurements. For determination of encapsulation efficiency, free decitabine in the supernatant was collected after centrifugation at 82,508 xg for 30 minutes at 4°C and measured by HPLC. The encapsulation efficacy of decitabine was defined as the mass ratio between the amount of drugs incorporated in liposomes and that used in the liposome preparation.

### Controlled release study of liposomes formulated decitabine

A controlled release study was performed using dialysis tubing (regenerated cellulose tubing, Mw cut-off 12000, 43 mm flat width, Crown Scientific, Australia) incubated in phosphate buffered saline (PBS) at 37°C. A 0.25 mL decitabine liposome suspension was added to the dialysis tubing immersed in a beaker with 10 mL of PBS as the release medium. Aliquots of 0.1 mL were collected from the solution outside the dialysis tubing at different time points. The volume of PBS was maintained by addition of 0.1 mL PBS after each withdrawal. The concentration of decitabine in each sample was determined using HPLC.

### Statistical analysis

Data were analyzed by GraphPad Prism (GraphPad Software, Inc.) using unpaired two-tailed t-tests, and linear and nonlinear regression.

## Results

### Development of a cell-based assay system EPISSAY for screening epigenetic drugs

**Figure 1:**
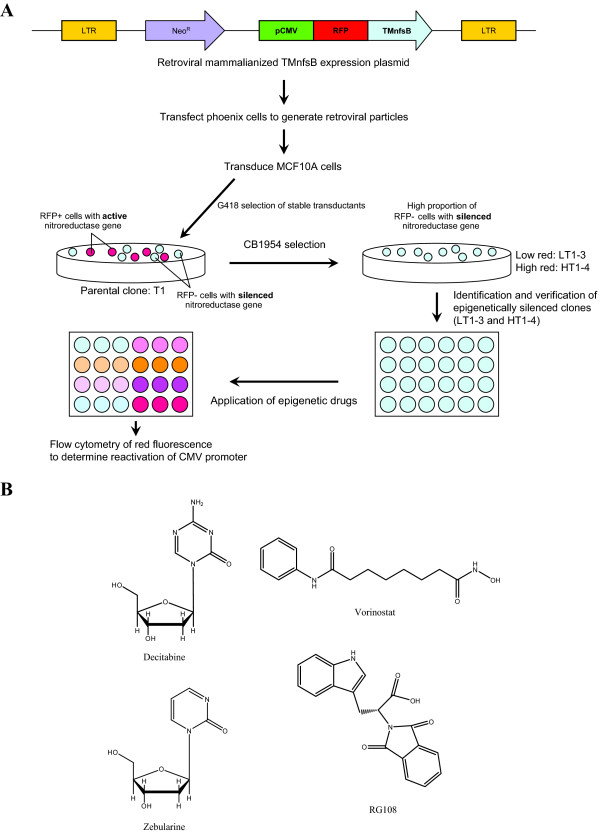
**The proposed EPISSAY system. **(**A**) Schematic showing different steps in development of the cell-based assay system for testing efficiency of epigenetic drugs. (**B**) Chemical structure of the epigenetic drugs used in this study.

The triple-mutated mammalianized version of *nfsB*, *TMnfsB*[26], was selected for developing the assay system as it showed the highest sensitivity to the lethal effect of CB1954 (Additional file 2). The schematic of the development of cell-based assay system for gene reactivation is in Figure 1. A stable MCF10A clone (T1) was generated which expressed the cytomegalovirus (CMV) promoter driven RFP-TMnfsB fusion protein (confirmed by western blot analysis, *data not shown*).

**Figure 2:**
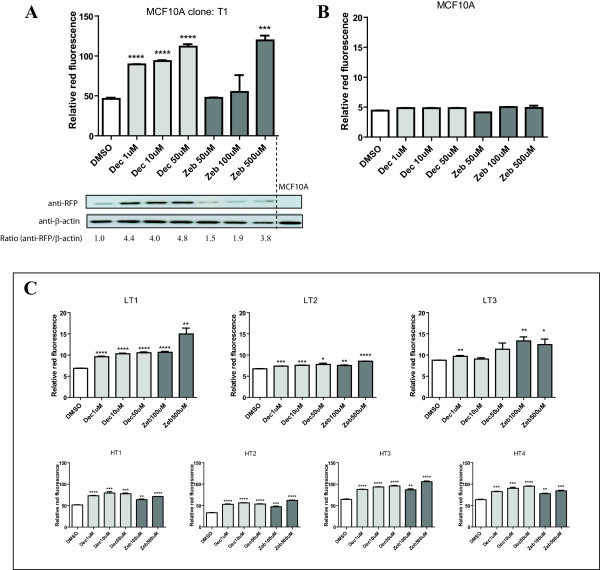
**Selection for EPISSAY system. **Flow cytometric assessment and western blot of the parental (**A**) *RFP-TMnfsB *expressing clone T1 and (**B**) untransduced MCF10A cells. The densitometry on western blots was quantified using ImageJ program. (**C**) Flow cytometric assessment of the CB1954-resistant clones generated from T1. Top panel: low fluorescent clones LT1, LT2 and LT3. Bottom panel: high fluorescent clones HT1, HT2, HT3 and HT4. Treatments were: decitabine 1, 10, 50 μM or zebularine 50, 100, 500 μM for 72 hours in triplicate in <1% v/v DMSO. Red-fluorescent reading is the gated geometric mean value of FL3-H. Note the different y axis scales for each panel. Unpaired two-tailed *t*-test, data expressed as mean ± SEM. * = p < 0.05, ** = p < 0.01, *** = p < 0.001, **** = p < 0.0001.

The CMV promoter is known to be gradually silenced over a period of months in culture and can be reactivated by subsequent treatment with epigenetic drugs [23]. Following growth of T1 for over two months there was increased expression of RFP-TMnfsB fusion protein after treatment with DNMT inhibitors (decitabine and zebularine) by western blot and flow cytometry analyses (Figure 2A). We observed that this was not due to auto-fluorescence of basal MCF10A cells (Figure 2B). This confirmed that the increased of red-fluorescent reading in clone T1 contained cells is due to the reactivation of silenced *RFP-TMnfsB*.

To identify the optimum clone for the basis of the assay system, cells of the T1 clone were treated with CB1954 to kill RFP positive cells which were expressing RFP-TMnfsB. Surviving clones will include those where the CMV promoter was silenced. These were screened for sensitivity to treatment with DNMT inhibitors (Figure 2C). Despite differences in the base levels of red-fluorescence, the red-fluorescent signals of all clones increased after treatment with decitabine and zebularine with clone LT1 showed the highest sensitivity.

### Proof of principle of the assay system

**Figure 3:**
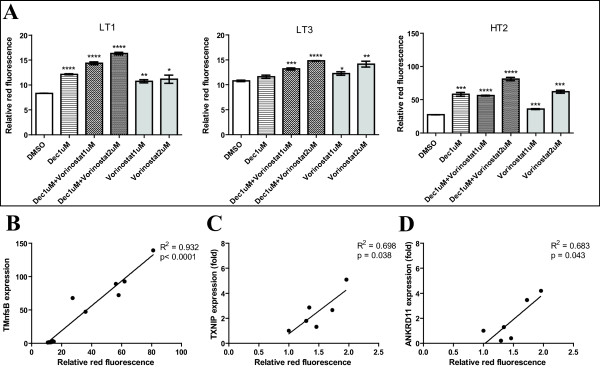
**Proof of principle of the assay system. **(**A**) Flow cytometric assessment of CB1954-resistant clone expressing *RFP-TMnfsB*. HT2, LT1, and LT3 were treated with 1 μM decitabine and/or 1, 2 μM of vorinostat (SAHA) for 48 hours. The average red-fluorescence of the treated cells (n = 3) were correlated with the mRNA expression of (**B**) *TMnfsB *of treated HT2 and LT3 (**C**) *TXNIP *and (**D**) *ANKRD11 *of treated LT1 cells normalized to *β-actin *expression (n = 1). The red-fluorescent reading for *TXNIP *and *ANKRD11 *analysis was normalized to vehicle control. All treatments contain <1% v/v of DMSO.

Three clones, LT1, LT3 and HT2, selected for additional testing were treated with decitabine and/or vorinostat for 72 hours, with media changes every 24 hours to maintain drug levels. An increased level of red-fluorescence was observed after treatment in all three clones (Figure 3A). Since the red-fluorescent signal should reflect expression of the *RFP-TMnfsB* gene, levels of *TMnfsB* mRNA were quantified in the treated cells (Figure 3B). There was a significant correlation between levels of red-fluorescence and *TMnfsB* expression in the clones with low and high initial red-fluorescence, LT3 and HT2, treated with decitabine and/or vorinostat (p < 0.0001), confirming that the red-fluorescent signal is directly related to the levels of *TMnfsB* message.

Among these clones, LT1 showed a lower red-fluorescent background and reasonable sensitivity to treatment with epigenetic drugs (Figures 2 and 3). To further validate these findings, the reactivation of two independent endogenous target genes was also assayed. The genes chosen were *TXNIP* (thioredoxin interacting protein) and *ANKRD11* (ankyrin repeat domain 11 protein), which were previously shown to be reactivated after treatment with decitabine and/or vorinostat [5,33,34]. The amount of *TXNIP* and *ANKRD11* in the LT1 cells was assessed after treatment with decitabine and/or vorinostat. A linear relationship of red-fluorescence and *TXNIP* mRNA expression (p < 0.05), and *ANKRD11* mRNA expression (p < 0.05), was observed (Figure 3C and 3D). Taken together, our data clearly show that increases in the levels of red-fluorescence signal are directly correlated with the endogenous *TXNIP* and *ANKRD11* reactivation in the cells treated with epigenetic drugs. We have named LT1 clone as EPISSAY and selected it for screening the activity of epigenetic drugs. We used EPISSAY to determine the effectiveness of a liposomal formulation of decitabine and to compare the existing epigenetic drugs.

### Development of liposomal formulated decitabine

Decitabine is an unstable compound that undergoes hydrolysis [32] and degradation by cytidine deaminase [35]. To improve the stability and bioavailability of decitabine, we formulated decitabine loaded liposomes by thin-film hydration as multilamellar liposomes with a broad size distribution of 871 ± 69 nm (Table 1). A narrow size distribution of decitabine-loaded liposomes was obtained by extruding the suspension through 400 nm and 200 nm filters to achieve a size of 138 ± 5 nm as unilamellar liposomes. The polydispersity index (PDI) of these extruded liposomes was less than 0.5 of the scale of 1 and liposomal formulation achieved an encapsulation efficacy of 55.1 ± 3.4% (0.15 μg decitabine/mg of lipid). The zeta potential of decitabine-loaded liposomes before extrusion was similar to the empty liposomes. The zeta potential of decitabine-loaded liposomes before extrusion -69.9 ± 2.8 increased to -40.2 ± 4.3 mV after extrusion. Overall the physiochemical data confirmed the decitabine-loaded liposomes are highly dispersed and achieved a smaller size <150 nm after extrusion. The potency of these newly formulated decitabine-loaded liposomes was subsequently compared with the free drug using the EPISSAY system.

**Table 1:** The physiochemical characteristics of the liposomal formulated decitabine

**Sample**	**Mean diameter, nm (± SD)**	**PDI**	**Zeta potential, mV (± SD)**
Decitabine-loaded liposomes	871 ± 69	0.358	-69.9 ± 2.8
Decitabine-loaded liposomes (E)	138 ± 5	0.296	-40.2 ± 4.3
Drug-free liposomes (MLVS)	1070 ± 77	0.744	-60.6 ± 2.7
Drug-free liposomes (E)	146 ± 1.6	0.137	-56.8 ± 0.9

*E: unilamellar liposomes extruded using 200 and 400 nm polycarbonate membranes.

PDI: polydispersity index.

MLVs: multilamellar vesicles.

### Use of EPISSAY system to determine the potency of liposomal formulated decitabine

**Figure 4:**
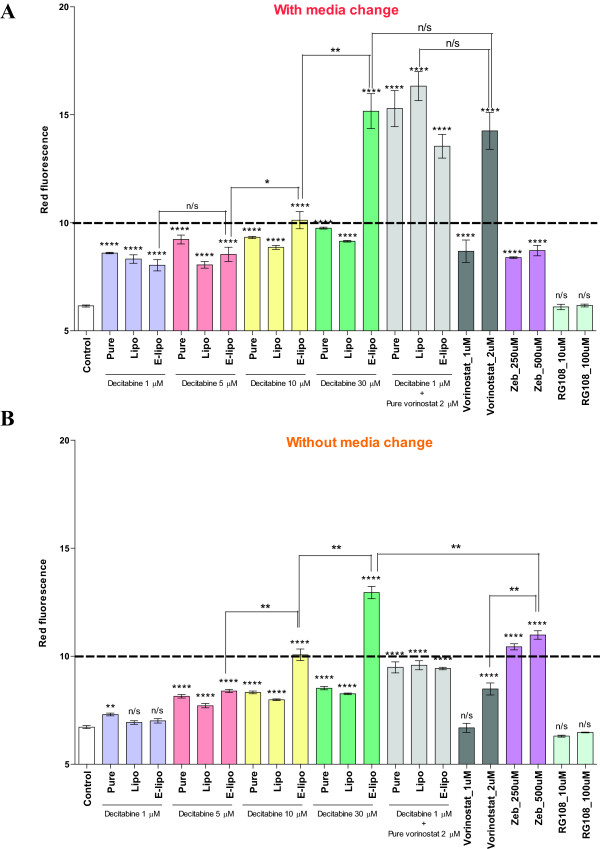
**The comparison of pure and newly-formulated epigenetic drugs using EPISSAY. **Flow cytometric assessment of LT1 cells treated with epigenetic drugs. Treatments were: liposomal formulated or pure decitabine 1, 5, 10, 30 μM and/or pure vorinostat 1, 2 μM; pure zebularine 250, 500 μM; pure RG108 10, 100 μM (**A**) with or (**B**) without media change for 72 hours in triplicate. The gated geometric mean values of FL3-H (red-fluorescence) were normalized to the vehicle control, drug-free liposomes and water. Lipo: multilamellar decitabine-loaded liposomes; E-lipo: unilamellar decitabine-loaded liposomes. Pure: drug without modification. Unpaired two-tailed *t*-test, data expressed as mean ± SEM. * = p < 0.05, ** = p < 0.01, *** = p < 0.001, **** = p < 0.0001.

To compare the potency of a panel of epigenetic drugs and newly formulated decitabine, LT1 cells were treated with these drugs for 72 hours, with or without a media change with fresh drug every 24 hours. Continuous treatment is often required as genes can be re-methylated after the removal of decitabine [36]. With a media change, 2 μM vorinostat and unilamellar decitabine-loaded liposomes at 30 μM were found to be more potent than pure decitabine and zebularine (Figure 4A). Notably, we observed a linear dose-dependent response in cells treated with unilamellar decitabine-loaded liposomes from 5 to 30 μM. There is a 50% increase of potency of the unilamellar decitabine-loaded liposomes compared with pure decitabine at 30 μM. In both with and without a media change, no significant difference was observed between treatment with 2 μM vorinostat alone and in the presence of 1 μM decitabine.

To investigate whether liposomal formulation protects decitabine from degradation, LT1 cells were treated with different concentrations of decitabine and liposomal decitabine for 72 hours without a media change (Figure 4B). A study of the drug release profile showed that 50% of decitabine was released from both unilamellar and multilamellar liposomes at ~90 minutes (Additional file 3). At 4 hours, the release of decitabine from unilamellar liposomes was slower (65%) than multilamellar liposomes (80%), confirming the better potency of unilamellar decitabine observed in Figure 4.

The potency of multilamellar decitabine-loaded liposomes and pure decitabine without media change were lower than those with the media change (Figure 4A and B). Nevertheless, the potency of unilamellar decitabine-loaded liposomes (10 μM) was maintained. Although unilamellar decitabine-loaded liposomes (30 μM) have the highest potency without media change, this was slightly reduced in comparison with replacing the drug every 24 hours. Taken together, our data showed that the potency of decitabine is improved when delivered as a unilamellar liposomal formulation.

## Discussion

EPISSAY, a cell-based assay system for screening of epigenetic drugs was developed based on the human non-malignant breast epithelial cell line MCF10A expressing the well-characterized CMV promoter driving RFP fused with a mammalianized version of the bacterial nitroreductase *nfs* gene. The *nfs* gene has been used in gene-directed enzyme prodrug therapy [37] since treatment of mammalian cells expressing *nfs* with CB1954 results in its chemical reduction to cytotoxic metabolites. Exposure of the derivative MCF10A with CB1954 was used as a strategy for the selection of cell lines with silenced *nfs* genes.

The EPISSAY was verified by treatment with the known epigenetic drugs decitabine, zebularine or vorinostat; all of which resulted in increased red-fluorescence due to reactivation of the CMV promoter. There was a linear relationship between *nfs* expression and the red-fluorescent signal confirming that levels of gene message and translated protein are directly related. The response was further confirmed by measuring expression levels of known independent endogenous genes *TXNIP*[5] and *ANKRD11*[34].

EPISSAY could be a time-saving assay for screening compounds with gene reactivating activity. Standard methodologies used to assess epigenetic compounds are based on quantitative real-time PCR and western blot analysis of genes known to be silenced in a particular cell line. For example, quantification of the re-expression of an endogenous gene p16 in human T24 bladder carcinoma cell line was previously used [38]. Such approaches are time-consuming as they require cell collection for RNA and protein extractions prior to analysis. Other cell-based assay systems which use exogenous expression of genes (e.g. *Escherichia coli* β-D-galactosidase gene with and green fluorescent reporter) have previously been investigated for their potential in screening epigenetic drugs by using fluorescent microscopy and plate readers. However, these other systems have limitations such as the non-quantitative data obtained and/or additional sample treatments required (e.g Paraformaldehyde fixing, the addition fluorogenic compounds) prior to screening [18-20] (Additional file 4). EPISSAY requires limited sample preparation, may be formatted for multi-well plates, and rapid results can be generated from RFP reading using flow cytometry to obtain quantitative data.

Decitabine is a demethylating agent that is FDA approved as an anti-cancer agent [13]. Since decitabine is degraded *in vivo* with a half-life of only 25 minutes, daily treatments are required to maintain appropriate drug levels both *in vitro* and *in vivo*[39]. To improve the stability and bioavailability of decitabine, the drug was encapsulated in PEGylated liposomes, as liposomes are known to protect drugs from degradation and allow controlled release of drug into the environment [40]. This formulation achieved an encapsulation efficiency of ~50%. Only 3.3 mol% of PEG 2000 was used in this study as a higher PEG content is known to reduce adsorption of liposomes onto cells [41].

Liposomes were extruded through filters with defined pore size (200 nm and 400 nm) to obtain unilamellar liposomes. Although extrusion does not affect the encapsulation efficiency [42], it narrowed the size distribution of the liposomes from 1 μm to approximately 150 nm. The smaller size of the drug-loaded liposomes has been reported to passively targeting disease tissues due to their enhanced angiogenesis [43].

We used the EPISSAY system to determine if liposomal encapsulation enhanced the gene reactivating activity of decitabine. Following 72 hours of treatment, decitabine encapsulated in unilamellar liposomes showed 50% more potency than pure decitabine, suggesting that decitabine was protected in the liposomes and slowly released into the media. These results were supported by a controlled release study comparing the drug release of decitabine from unilamellar and multilamellar liposomes. This showed that the release rate of decitabine from unilamellar liposomes was slower, suggesting unilamellar liposomal formulation may decrease the rate of degradation of decitabine by providing protection to the drug. In addition, the liposomal formulation and the presence of phospholipids in the cell media could also contribute to the enhancement of decitabine activity [44,45].

Collectively, the liposomal decitabine that was synthesised here was validated as a more potent epigenetic drug. However, we have only confirmed this *in vitro*. An *in vivo* study of liposomal decitabine is recommended to assess its applicability for clinical use, and to confirm if the present limitations of decitabine use in the clinic could be overcome by this formulation. The use of liposomes/PEG to encapsulate drugs to improve their bio-availability and stability is now gaining momentum with a number of drugs eg doxorubicin [17], rhenium radionuclides [46] and dexamethasone phosphate [47], liposome-encapsulated doxorubicin now having FDA approval.

## Conclusions

In this pilot study, we have constructed and evaluated a novel bioassay for epigenetic compounds. The readout of the EPISSAY system is red-fluorescence, which may allow the adaptation of the assay system to a multi-well format allowing high throughput, rapid, and cheap bioassay in the future. EPISSAY was successful in providing evaluation of different liposomal formulations of decitabine. The EPISSAY can detect the gene reactivating effects of decitabine, zebularine or vorinostat. Linear correlation between the message of an endogenous gene *ANKRD11* and red-fluorescent reading has been shown in the EPISSAY cells treated with pure decitabine and unilamellar liposomes-formulated decitabine (Additional file 5).

Using SEQUENOM MassARRAY EpiTYPER, no major changes in methylation of the CMV promoter was detected in the EPISSAY cells before and after treatments with decitabine (Additional file 6 and 7). Although vorinostat is known as a HDAC inhibitor to activate gene expression, zebularine and decitabine are usually considered to function as demethylating agents or DNMT inhibitors [48]. However, there are now multiple studies that show these agents can also function as HDAC inhibitors [49-51]. This suggests that the *TMnfsB* gene was most likely silenced by histone modification rather than direct methylation of the CMV promoter. There is a potential of adopting this assay as a high throughput, rapid and low cost epigenetic drug screening platform are unique aspects of the EPISSAY system. We conclude that our EPISSAY bioassay system provides a novel and rapid system to screen the efficiencies of epigenetic and newly formulated drugs for gene reactivation.

## Competing interests

The authors declare that they have no competing interests.

## Authors’ contributions

SPL carried out all the experimental work and drafted the manuscript. RK carried out the molecular biology studies, participated in the experimental design and contributed to drafting and editing of the manuscript. YA and WW participated in the study of nanotechnology. KH, PMN and DJW contributed to the molecular biology studies. RJS was involved in the design of the study, performed the statistical analysis and edited the manuscript. CP reviewed the study and participated in the nanotechnology work. DFC supervised the study, and contributed to its design and coordination and helped to draft the manuscript. All authors read and approved the final manuscript.

## Pre-publication history

The pre-publication history for this paper can be accessed here:

http://www.biomedcentral.com/1471-2407/13/113/prepub

## Supplementary Material

Additional file 1 PCR primers used in this study. Click here for file

Additional file 2 **Sensitivity of different nitroreductase genes to CB1954. **Transiently transfected HEK293T cells with (**A**) pDsRED-monomer-C1 vector, (**B**) pDsRED-nfsA, (**C**) pDsRED-nfsB, (**D**) pDsRED-MnfsB, (E) pDsRED-TMnfsB and incubated with 0, 1, 5, 10 μM of CB1954 for 24 hours at 37°C/ 5% CO_2_. All contain 0.2% v/v DMSO. The decreased of red-fluorescence indicates cell death. Click here for file

Additional file 3 **Controlled release study of liposomal decitabine. **(**A**) The standard plot of pure decitabine produced using HPLC at 254 nm (retention time = 6.554 ± 0.003 minutes). (**B**) Drug release profiles of unilamellar and multilamellar liposomal decitabine at different time intervals generated using the standard plot of pure decitabine. Click here for file

Additional file 4 Characteristics of previously investigated epigenetic cell-based assay systems. Click here for file

Additional file 5 **The correlation of endogenous *ANKRD11 *expression and the relative red-fluorescence in the EPISSAY system. **The average red-fluorescence of the treated cells (n=3) were correlated with the mRNA expression of *ANKRD11* (n=1). The EPISSAY (LT1) cells were treated with 1, 5, 10, 30 μM of pure decitabine and unilamellar liposomes-formulated decitabine for 72 hours with/ without a media change every 24 hours to replenish the level of drugs. *ANKRD11 *of treated LT1 cells was normalized to *β-actin *expression. The red-fluorescent reading was normalized to vehicle control. Click here for file

Additional file 6 **Epigram showing methylation levels of the CMV promoter generated from SEQUENOM EpiTYPER Platform. **This epigram showed % CpG methylation of CMV promoter in overlapping regions of CMV_1 and CMV_2 amplicons of RFP-TMnfsB expressing clones treated with epigenetic drugs are indicated (n=2). Dec: decitabine; Zeb: zebularine. LT1 is the CB1954-resistant clone, which subsequently in used as the basis of EPISSAY. T1 is the parental clone without CB1954 selection and has a higher red-fluorescent background than LT1. The CpG units are as defined in Addition file 7. Click here for file

Additional file 7 **Amplicon design and the target region for methylation analysis.** Bisulfite treated sequence of CMV promoter regions: CMV_1; CMV_2. [T bold: cytosine from non-CG converted to T; italic smaller font: primer target sequence; all CGs: bold; CG underlined: analysed CGs; |Unit|: fragment with different mass and size generated by enzymatic base specific cleavage]. Click here for file
