# Supplementary material for: Development of a novel cell-based assay system EPISSAY for screening epigenetic drugs and liposome formulated decitabine
Source: BMC Cancer. 2013 Mar 13;13:113. doi: 10.1186/1471-2407-13-113 (PMC3637807; doi:10.1186/1471-2407-13-113)
Supplement: Additional file 2 — Sensitivity of different nitroreductase genes to CB1954. Transiently transfected HEK293T cells with (A) pDsRED-monomer-C1 vector, (B) pDsRED-nfsA, (C) pDsRED-nfsB, (D) pDsRED-MnfsB, (E) pDsRED-TMnfsB and incubated with 0, 1, 5, 10 μM of CB1954 for 24 hours at 37°C/ 5% CO2. All contain 0.2% v/v DMSO. The decreased of red-fluorescence indicates cell death. [file 1471-2407-13-113-S2.doc]

Additional file 2


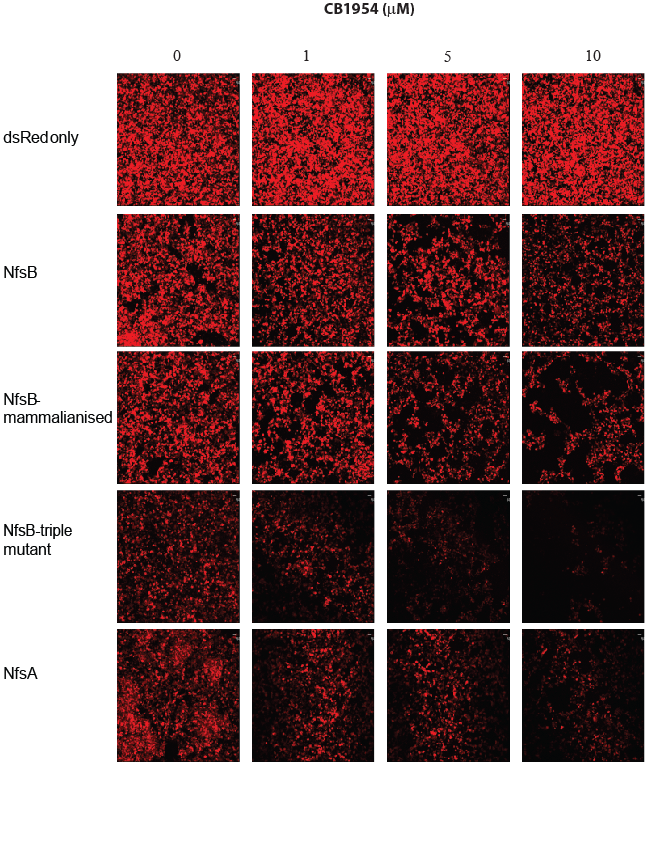


**Note:**

To select the most sensitive version of the nitroreductase (*nfs*) gene, the sensitivities of different bacterial *nfs* genes including wild-type *nfsA*, wild-type *nfsB*, mammalianised *nfsB* (*MnfsB*) and triple-mutated mammalianised *nfsB* (*TMnfsB*) towards CB1954, were tested in transiently transfected HEK293T cells. In the result, there was a trend of increased cell death in *nfs* expressing cells with increasing concentration of CB1954. The cells without *nfs* showed no cell death, confirming the selectivity of the treatment towards cells expressing *nfs*. Cells expressing the mammalianised version of *nfsB* have a higher sensitivity to CB1954 than either *nfsA* or *nfsB*. Among them, cells expressing the triple-mutated mammalianised version of *nfsB*, *TMnfsB*, possessed the highest sensitivity toward CB1954 and was selected as the basis for the assay system.
