# Supplementary material for: Development of a novel cell-based assay system EPISSAY for screening epigenetic drugs and liposome formulated decitabine
Source: BMC Cancer. 2013 Mar 13;13:113. doi: 10.1186/1471-2407-13-113 (PMC3637807; doi:10.1186/1471-2407-13-113)
Supplement: Additional file 4 — Characteristics of previously investigated epigenetic cell-based assay systems. [file 1471-2407-13-113-S4.doc]

| Screening method used | Epigenetic drugs tested | Preparation required prior screening | Promoter gene to drive the reporter system | Reporter system | Cell line | Limitations | Ref. |
| --- | --- | --- | --- | --- | --- | --- | --- |
| Flow cytometry | DNMT inhibitors | Addition of fluorescein di-β-D-galactopyranoside substrate | *Mouse Metallothionein 1 (mMT-1)* | *Escherichia coli* /β-galactosidase  coding gene (LacZ) | Human embryonic kidney, HEK293 | Additional treatment of sample required prior screening | [1] |
| Fluorescence microscopy | DNMT inhibitors | Paraformaldehyde fixing (4%) | *Endogenous FLJ32130* | Hygromycin-resistance fused with enhanced green fluorescent protein (Hyg^r^-EGFP) | Human colon cancer, HCT116 | Qualitative  green fluorescent background of the cells, weak promoter | [2] |
| Fluorometric plate reader | HDAC inhibitors | Addition of fluorogenic acetyl-lysine substrate | - | Deacetylated fluorogenic peptide product | Partially purified HeLa cell nuclear extract | Additional treatment of sample required prior screening | [3] |

**References of additional file 4:**

1. Biard DSF, Maratrat M, Thybaud Vr, et al. Flow cytometric detection of drugs altering the DNA methylation pattern. Cancer Research 1992;52 (19):5213-18.

2. Okochi-Takada E, Ichimura S, Kaneda A, et al. Establishment of a detection system for demethylating agents using an endogenous promoter CpG island. Mutation Research/Fundamental and Molecular Mechanisms of Mutagenesis 2004;568 (2):187-94.

3. Hassig CA, Symons KT, Guo X, et al. KD5170, a novel mercaptoketone-based histone deacetylase inhibitor that exhibits broad spectrum antitumor activity in vitro and in vivo. Molecular Cancer Therapeutics 2008;7 (5):1054-65.
