# Supplementary material for: Development of a novel cell-based assay system EPISSAY for screening epigenetic drugs and liposome formulated decitabine
Source: BMC Cancer. 2013 Mar 13;13:113. doi: 10.1186/1471-2407-13-113 (PMC3637807; doi:10.1186/1471-2407-13-113)
Supplement: Additional file 5 — The correlation of endogenous ANKRD11 expression and the relative red-fluorescence in the EPISSAY system. The average red-fluorescence of the treated cells (n=3) were correlated with the mRNA expression of ANKRD11 (n=1). The EPISSAY (LT1) cells were treated with 1, 5, 10, 30 μM of pure decitabine and unilamellar liposomes-formulated decitabine for 72 hours with/ without a media change every 24 hours to replenish the level of drugs. ANKRD11 of treated LT1 cells was normalized to β-actin expression. The red-fluorescent reading was normalized to vehicle control. [file 1471-2407-13-113-S5.doc]

Additional File 5

**Additional file 5** The correlation of endogenous *ANKRD11* expression and the relative red-fluorescence in the EPISSAY system. The average red-fluorescence of the treated cells (n=3) were correlated with the mRNA expression of *ANKRD11* (n=1). The EPISSAY (LT1) cells were treated with 1, 5, 10, 30 μM of pure decitabine and unilamellar liposomes-formulated decitabine for 72 hours with/ without a media change every 24 hours to replenish the level of drugs. *ANKRD11* of treated LT1 cells was normalized to* β-actin* expression. The red-fluorescent reading was normalized to vehicle control.
