# Supplementary material for: Development of a novel cell-based assay system EPISSAY for screening epigenetic drugs and liposome formulated decitabine
Source: BMC Cancer. 2013 Mar 13;13:113. doi: 10.1186/1471-2407-13-113 (PMC3637807; doi:10.1186/1471-2407-13-113)
Supplement: Additional file 6 — Epigram showing methylation levels of the CMV promoter generated from SEQUENOM EpiTYPER Platform. This epigram showed % CpG methylation of CMV promoter in overlapping regions of CMV_1 and CMV_2 amplicons of RFP-TMnfsB expressing clones treated with epigenetic drugs are indicated (n=2). Dec: decitabine; Zeb: zebularine. LT1 is the CB1954-resistant clone, which subsequently in used as the basis of EPISSAY. T1 is the parental clone without CB1954 selection and has a higher red-fluorescent background than LT1. The CpG units are as defined in Addition file 7. [file 1471-2407-13-113-S6.doc]

Additional File 6


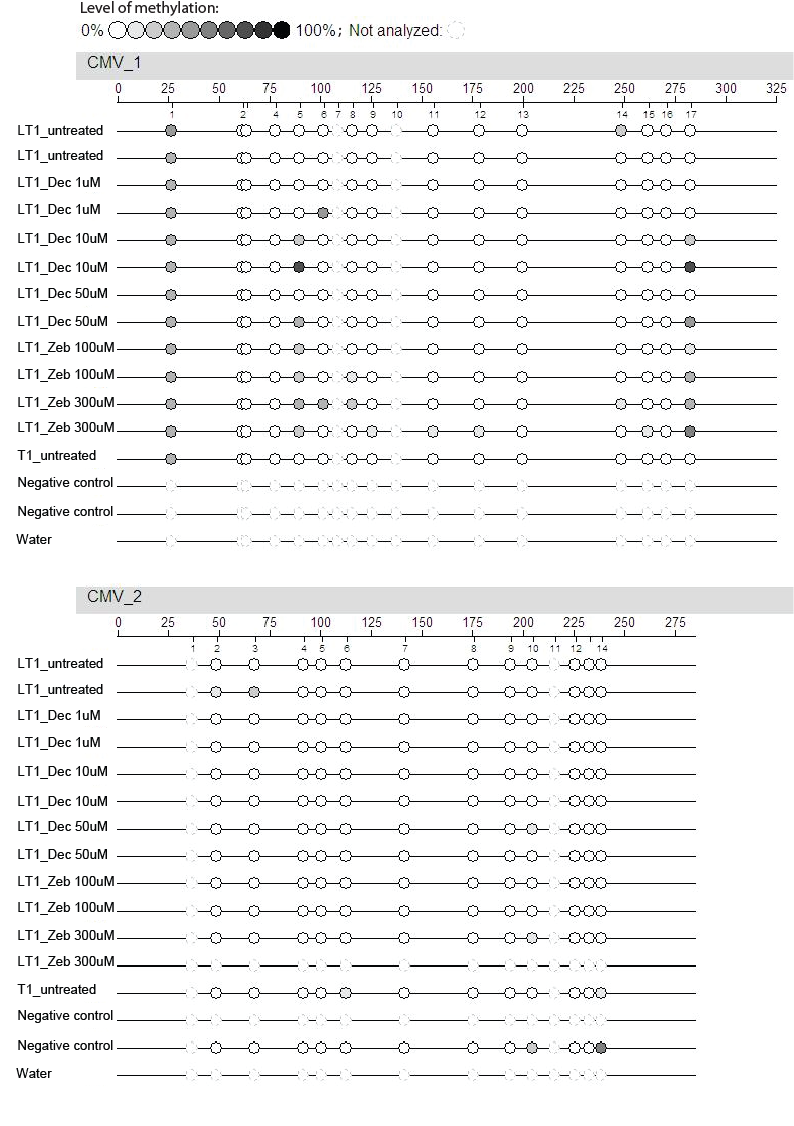


**Additional file 6** Epigram showing methylation levels of the CMV promoter generated from SEQUENOM EpiTYPER Platform. This epigram showed % CpG methylation of CMV promoter in overlapping regions of CMV_1 and CMV_2 amplicons of RFP-TMnfsB expressing clones treated with epigenetic drugs are indicated (n=2). Dec: decitabine; Zeb: zebularine. LT1 is the CB1954-resistant clone, which subsequently in used as the basis of EPISSAY. T1 is the parental clone without CB1954 selection and has a higher red-fluorescent background than LT1. The CpG units are as defined in Addition file 6.

***Results****:*

*The levels of red-fluorescence of LT1 (EPISSAY) cells were previously shown to increase following treatment with either zebularine or decitabine, suggesting possible demethylation of the CMV promoter in LT1 cells. Cells from treatments with demethylating agents were analysed for expression of RFP-TMnfsB and for CMV promoter methylation. The T1 (parental clone) and LT1 did not demonstrate consistent differences in methylation across the CMV promoter. The samples analysed following treatments with demethylating agents showed inconsistent levels of methylation at some sites. These data suggest gene silencing in these cells was not due to DNA methylation.*

#### *Material and method:*

CMV promoter is responsible for driving the transcription of RFP-TMnfsB in the cell-based assay system. To analyse the methylation level of the CMV promoter, DNA samples were bisulphite-treated and purified using the Epitect Kit (Qiagen) according to the manufacturer’s protocol. Unconverted genomic DNA was included as a negative control. *In vitro* transcription and uracil-specific cleavage of the amplified products were undertaken before analysis of the samples by matrix-assisted laser desorption and ionisation time-of-flight mass spectrometry (MALDI-TOF-MS; SEQUENOM EpiTYPER Platform) as previously described ^[34]^. Two overlapping regions (CMV_1 and CMV_2) of the bisulfite-treated CMV promoter sequence were amplified using the PyroMark Kit (Qiagen) and primers given below. Lowercase denotes sequence tags added to facilitate downstream EpiTYPER analysis.

CMV_1:

5′-AGGAAGAGAGAATAGTAATTAATTACGGGGTTATTAGTTTATAGTTT-3′ 5′-CAGTAATACGACTCACTATAGGGAGAAGGCccataaaatcatatactaaacataata-3′

CMV_2:

5′-AGGAAGAGAGATTTTTTTATTTGGTAGTATATTTACGTATTAGTT-3′

5′-CAGTAATACGACTCACTATAGGGAGAAGGCttcactaaaccaactctacttatataa-3′
