# Supplementary material for: Development of a novel cell-based assay system EPISSAY for screening epigenetic drugs and liposome formulated decitabine
Source: BMC Cancer. 2013 Mar 13;13:113. doi: 10.1186/1471-2407-13-113 (PMC3637807; doi:10.1186/1471-2407-13-113)
Supplement: Additional file 7 — Amplicon design and the target region for methylation analysis. Bisulfite treated sequence of CMV promoter regions: CMV_1; CMV_2. [T bold: cytosine from non-CG converted to T; italic smaller font: primer target sequence; all CGs: bold; CG underlined: analysed CGs; |Unit|: fragment with different mass and size generated by enzymatic base specific cleavage]. [file 1471-2407-13-113-S7.doc]

Additional File 7

**
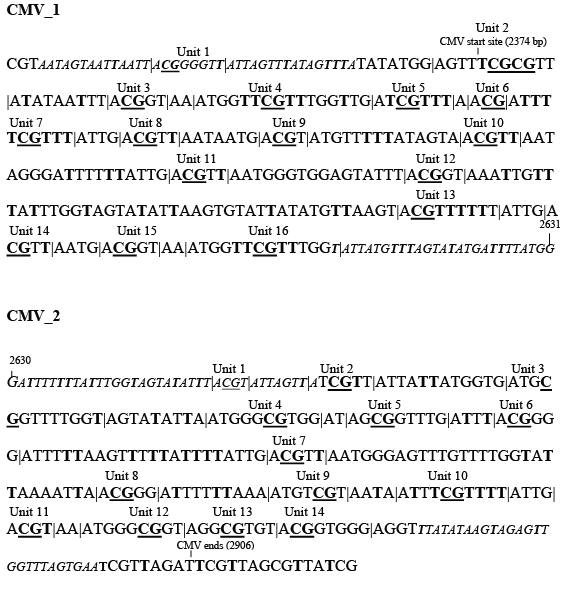
**

**Additional file 7** Amplicon design and the target region for methylation analysis. Bisulfite treated sequence of CMV promoter regions: CMV_1; CMV_2. [T bold: cytosine from non-CG converted to T; *italic smaller font*: primer target sequence; all CGs: bold; CG underlined: analysed CGs; |Unit|: fragment with different mass and size generated by enzymatic base specific cleavage.]
